# Investigation of Ochratoxin A Levels in Commercially Available Turkish Coffee and Risk Assessment

**DOI:** 10.3390/toxins18020084

**Published:** 2026-02-06

**Authors:** Hayrettin Özer

**Affiliations:** TÜBİTAK Marmara Araştırma Merkezi, 41470 Gebze, Kocaeli, Türkiye; hayrettin.ozer@tubitak.gov.tr; Tel.: +90-532-554-5585

**Keywords:** Turkish coffee, ochratoxin A, risk assessment

## Abstract

This study evaluated the occurrence of ochratoxin A (OTA) in Turkish coffee and its potential health implications under current consumption patterns by analyzing 65 ground and roasted Turkish coffee samples collected across Türkiye. OTA contamination was detected in 53 samples (82%). Based on the mean OTA concentration, the Estimated Daily Intake (EDI) was calculated as 0.1403 ng/kg body weight/day, and health risk characterization was performed using the Margin of Exposure (MOE) approach in accordance with the European Food Safety Authority (EFSA) recommendations for chronic exposure assessment. MOE calculations enabled a refined characterization of health risks under realistic (0.5 cup/day), average (1 cup/day), and high (3 cups/day) consumption scenarios. The MOE values for carcinogenic (neoplastic) effects ranged from 34,450 to 206,847, all exceeding the EFSA reference threshold of 10,000 and indicating a low level of concern for carcinogenic risk associated with Turkish coffee consumption. For non-carcinogenic (non-neoplastic) kidney effects, MOE values ranged from 11,238 to 67,475 across the different consumption scenarios, all exceeding the EFSA reference threshold of 200, indicating a low level of concern for the general population. In conclusion, the findings demonstrate that Turkish coffee consumption does not pose an OTA-related carcinogenic or non-neoplastic health risk for the general population under current consumption patterns. Nevertheless, considering the widespread consumption of Turkish coffee, continued monitoring and strict implementation of control measures throughout the production chain remain advisable to ensure long-term consumer safety.

## 1. Introduction

OTA is a mycotoxin of major public health concern due to its frequent occurrence in food commodities and its well-documented nephrotoxic and carcinogenic potential [1,2]. Among dietary sources, coffee has been identified as a relevant contributor to human OTA exposure because of its widespread consumption and the relative stability of OTA during processing [3,4]. Several surveys and market studies have reported the presence of OTA in coffee beans and roasted coffee products, indicating that coffee may represent a continuous low-level exposure source for consumers [5,6]. Given the high global consumption frequency of coffee, even low concentrations of OTA may be relevant from a risk assessment perspective and contribute meaningfully to overall dietary exposure [4].

Coffee is a globally traded agricultural commodity of high economic value, and contamination with OTA caused by toxigenic fungal growth represents a major constraint affecting coffee quality, safety, and international trade [7,8]. OTA contamination may occur at multiple stages of post-harvest processing, particularly under inadequate drying, storage, and transportation conditions that favor fungal growth [3,9] OTA contamination in coffee has been primarily associated with ochratoxigenic Aspergillus species, including *A. ochraceus*, *A. westerdijkiae*, *A. carbonarius*, and members of the *A. niger* species complex [5,10].

Several market surveys conducted in Türkiye over different years have reported the presence of OTA in coffee products, including Turkish coffee. However, substantial variability has been observed in both detection frequency and reported OTA concentrations, largely depending on coffee type, sampling year, and analytical methodology. This heterogeneity complicates direct comparison across studies and highlights the need for updated and systematically generated data on OTA occurrence in commercially available Turkish coffee. Previous national studies reporting OTA occurrence in coffee products marketed in Türkiye are summarized in Supplementary Table S1.

In this study, the term “Turkish coffee” refers exclusively to the traditional Turkish method of coffee preparation and consumption and does not indicate the geographical origin of the coffee beans, which are imported, as Türkiye does not produce coffee. Global coffee consumption has increased steadily over recent decades, and this trend is also evident in Türkiye [11]. Changes in consumer habits, increased product availability, and diversification of coffee types have contributed to higher consumption frequencies [12]. Within this expanding market, Turkish coffee holds a distinctive position due to its unique preparation method, cultural significance, and consumption habits. Recognized by UNESCO as an element of the Intangible Cultural Heritage of Humanity since 2013 [12,13], Turkish coffee remains the most preferred coffee type in Türkiye despite changing consumption trends [14]. Survey-based studies have consistently reported frequent and regular consumption of Turkish coffee across different regions and demographic groups, with a substantial proportion of consumers reporting weekly or daily intake [12,14,15]. Given the widespread and habitual consumption of Turkish coffee, even low-level OTA contamination may contribute meaningfully to dietary exposure, underscoring the importance of evaluating OTA occurrence and associated health risks in this specific coffee type. Turkish coffee is prepared by mixing finely ground roasted coffee with water, gradually heating it to boiling, often with repeated short boiling cycles, and serving it unfiltered with the coffee grounds remaining in the cup. This preparation method involves prolonged contact between water and coffee particles under high-temperature conditions, which may influence the transfer of ochratoxin A (OTA) from ground coffee into the beverage.

Experimental studies indicate that OTA is not fully transferred during Turkish coffee preparation. Santini et al. reported that Turkish coffee prepared from OTA-spiked roasted coffee showed an OTA transfer of approximately 42–50% into the beverage, while none of the evaluated brewing methods resulted in complete OTA transfer and maximum extraction did not exceed 81% [16]. Similarly, Malír et al. demonstrated that only about 51–54% of naturally occurring OTA present in roasted and ground coffee was transferred into Turkish coffee prepared by repeated boiling [17]. Earlier qualitative observations by La Pera et al. also indicated relatively high OTA extraction during Turkish coffee preparation compared with shorter-contact brewing methods [18].

Moreover, Turkish coffee is traditionally consumed unfiltered, with fine coffee particles remaining in suspension and potentially ingested together with the beverage, which further supports the use of a conservative, worst-case assumption in dietary exposure assessment. Accordingly, assuming complete OTA transfer from ground coffee to the beverage represents a protective approach that is unlikely to underestimate consumer exposure. In response to the recognized public health risks associated with OTA exposure, regulatory authorities have established maximum allowable limits for this mycotoxin in food products. According to European Union (EU) legislation (Commission Regulation EC No. 2023/915) and the Turkish Food Codex Contaminants Regulation (Official Gazette No. 28157, 29.12.2011), the maximum permitted OTA levels are set at 5.0 µg/kg for roasted and ground coffee and 10.0 µg/kg for coffee extracts [19,20]. More recently, Regulation (EU) No. 2022/1370, effective from 2023, lowered the maximum OTA levels to 3 μg/kg for roasted and ground coffee and to 5 μg/kg for soluble coffee, reflecting increased awareness of OTA-related health concerns and the need for enhanced consumer protection. Given the widespread consumption of coffee and the growing global interest in Turkish coffee, robust exposure assessment and risk characterization are essential to ensure compliance with regulatory standards and to support effective risk management strategies. Within this regulatory context, health risk assessment frameworks such as the MOE approach are widely applied to support evidence-based risk characterization and consumer protection strategies [21].

Based on existing evidence indicating frequent but generally low-level occurrence of OTA in coffee products, it was hypothesized that OTA contamination in commercially available Turkish coffee would be common but would not pose a significant public health concern under typical consumption patterns. Accordingly, the aim of the present study was to evaluate the occurrence of OTA in commercially available Turkish coffee, to estimate dietary exposure under different consumption scenarios, and to characterize the associated public health risk using the MOE approach.

## 2. Results

**Figure 1:**
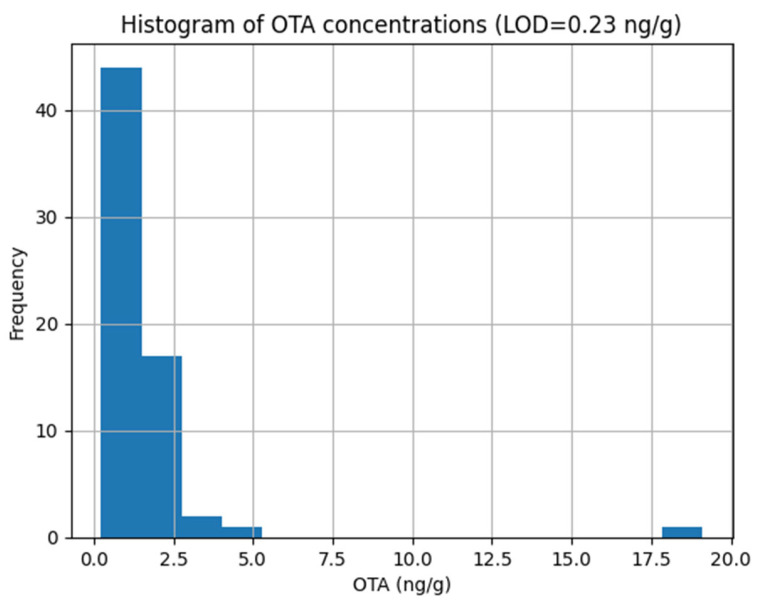
Histogram of OTA concentrations in Turkish coffee samples.

**Figure 2:**
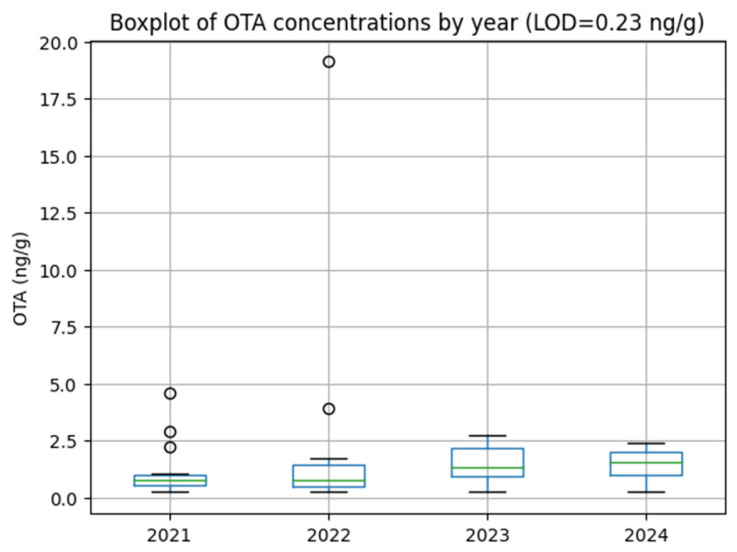
Boxplot of OTA concentrations in Turkish coffee samples. Sample sizes per year: 2021 (*n* = 18), 2022 (*n* = 18), 2023 (*n* = 14), and 2024 (*n* = 15).

OTA contamination was detected in 53 of the 65 Turkish coffee samples analyzed, corresponding to an overall positivity rate of 82%. The distribution of OTA concentrations is summarized in Table 1 and illustrated in Figure 1 and Figure 2. Only one sample exceeded the maximum level permitted under the Turkish Food Codex and EU regulations, while all remaining samples complied with the applicable regulatory limits.

Using the mean OTA concentration (1.473 ng/g) and three consumption scenarios—realistic (0.5 cup/day), average (1 cup/day), and high (3 cups/day)—EDI values were estimated at 0.0701, 0.1403, and 0.4209 ng/kg body weight/day, respectively (Table 2).

MOE values were calculated using the EFSA-recommended benchmark dose lower confidence limit for a 10% response (BMDL10). As shown in Table 2, MOE values for neoplastic effects ranged from 34,450 to 206,847 across the three consumption scenarios, while MOE values for non-neoplastic kidney effects ranged from 11,238 to 67,475. All calculated MOE values exceeded the EFSA reference thresholds of 10,000 for neoplastic effects and 200 for non-neoplastic effects.

The OTA concentration distribution showed a right-skewed pattern with one extreme value, indicating that mean exposure estimates may be influenced by occasional high-contamination events.

Despite the high detection frequency, the narrow interquartile ranges observed in most years suggest a relatively stable OTA contamination pattern, with occasional outliers rather than systematic exceedances.

## 3. Discussion

The results of this study demonstrate that OTA occurrence in Turkish coffee is common; however, contamination levels generally remain below regulatory limits. OTA has been classified by the International Agency for Research on Cancer (IARC) as a Group 2B compound, indicating possible carcinogenicity to humans, with nephrotoxicity identified as the primary non-neoplastic endpoint [22,23,24,25]. Against this toxicological background, the MOE approach applied in the present study provides a robust framework for interpreting the observed exposure levels in relation to established toxicological reference points. The high detection frequency observed is consistent with previous national and international studies reporting widespread but generally low-level OTA presence in coffee products.

The prevalence of OTA detected in the present study is consistent with previous reports highlighting the widespread occurrence of OTA in coffee products. Pérez-de-Obanos et al. (2005) demonstrated that the transfer of ochratoxin A from green coffee beans to the final drinking form is strongly influenced by roasting and brewing conditions, which in turn contributes to the variability observed among coffee samples [26]. Similarly, Vanesa and Ana reported OTA contamination in 35 out of 51 coffee samples including green, ground and roasted coffee, with concentrations ranging from 0.1 to 21.0 ng/g [27]. Casal et al. also detected OTA in 35 of 40 soluble ground coffee samples marketed in Portugal, with levels between 0.15 and 12.00 µg/kg [28]. Together, these findings indicate that while OTA occurrence in coffee is common, contamination levels are generally comparable across different markets and regions.

Although the overall OTA contamination levels observed in this study were largely within the regulatory limits, a single sample was found to exceed the maximum permitted level. This exceedance may be linked to factors such as suboptimal post-harvest handling, prolonged storage under high humidity, or compromised packaging integrity along the coffee supply chain. While no causal relationship can be established within the scope of this study, these steps represent potential critical control points for reducing OTA contamination.

Exposure assessment showed that OTA intake via Turkish coffee consumption is low for the general population. The calculated EDI values support the exposure assessment, while health risk characterization is appropriately addressed using the MOE-based approach., indicating that Turkish coffee is not a major contributor to overall OTA exposure in Türkiye.

Although the risk assessment framework has evolved toward the MOE approach, current data indicate that roasted and ground coffee represents a relevant dietary source of human OTA exposure [6,29]. A hypothetical exposure scenario based on the consumption of a single cup of coffee prepared with approximately 7 g of coffee containing 12 ng/g OTA illustrates that such intake levels are not negligible and may contribute to overall dietary exposure [30,31]. These considerations highlight the importance of controlling OTA levels in coffee products, particularly in the context of cumulative dietary exposure from multiple food sources.

Consistent with EFSA (2020) recommendations, MOE analysis showed that MOE values for both neoplastic and non-neoplastic effects exceeded the respective reference thresholds across all consumption scenarios, indicating a low level of public health concern under current Turkish coffee consumption patterns [2]. MOE values above the EFSA reference thresholds indicate that estimated dietary exposure remains sufficiently below the toxicological reference points and therefore does not raise a concern for human health. This finding is particularly relevant in light of the established nephrotoxic potential of OTA and its classification as a possible human carcinogen. Similarly, global exposure assessments have reported wide variability in MOE values for OTA across different countries, reflecting differences in consumption patterns and contamination levels [32].

It should also be noted that the exposure estimates were derived using conservative assumptions, including complete transfer and solubilization of OTA from ground coffee into the consumed beverage. In the specific context of Turkish coffee, which is traditionally consumed unfiltered with fine coffee particles remaining in the cup and potentially ingested together with the beverage, this worst-case assumption is considered appropriate and protective for public health. Under such consumption conditions, exposure may not be limited to the dissolved fraction of OTA in the liquid phase alone, thereby supporting the use of a conservative exposure scenario. At the same time, available experimental evidence indicates that OTA is not fully transferred during coffee preparation; therefore, actual consumer exposure is likely to be lower than the estimated values reported in this study.

These findings are in agreement with exposure assessments from other countries, where coffee consumption has been shown to contribute marginally to total OTA intake. Nevertheless, given the cultural importance and widespread consumption of Turkish coffee, continued monitoring remains important to ensure that OTA levels remain within safe limits and to support effective risk management strategies. From a risk management perspective, these findings underline the importance of effective control measures along the coffee supply chain in Türkiye. In particular, strengthened screening of raw coffee beans, strict control of storage conditions—especially humidity—and regular monitoring of packaging integrity represent critical control points to minimize OTA contamination and ensure consumer protection.

## 4. Conclusions

This study evaluated the occurrence of OTA in commercially available roasted and ground Turkish coffee samples from Türkiye, estimated dietary exposure, and characterized the associated public health risk using the MOE approach. OTA was detected in the majority of samples; however, contamination levels generally remained below the maximum limits established by both the Turkish Food Codex and current EU regulations, with only a single sample exceeding the applicable regulatory threshold. Exposure assessment and MOE analysis, conducted in line with EFSA recommendations, indicated a low level of public health concern for both neoplastic and non-neoplastic effects under current Turkish coffee consumption patterns. Although MOE values for non-neoplastic kidney effects were lower than those for carcinogenic outcomes, they remained above the relevant EFSA reference thresholds. Overall, these findings suggest that current OTA exposure through Turkish coffee consumption does not pose a significant public health risk, while continued monitoring remains important given the widespread consumption and cultural significance of Turkish coffee. Extended analyses directly quantifying ochratoxin A in Turkish coffee beverages would allow for further refinement of exposure estimates and risk characterization based on actual beverage intake, while the present results provide a conservative and protective assessment.

## 5. Materials and Methods

### 5.1. Reagents, Standards, and Solutions

All organic solvents used for mobile phase preparation and OTA standard solutions were of High-Performance Liquid Chromatography (HPLC) grade. Acetonitrile and methanol were obtained from Scharlau (Istanbul, Türkiye); acetic acid from Sigma-Aldrich (St. Louis, MO, USA); and ultrapure water from Millipore (Darmstadt, Germany). The reference OTA standard was purchased from Trilogy (Austin, TX, USA).

### 5.2. Test Material

All coffee samples were obtained exclusively from nationally distributed supermarket chains operating standardized procurement systems across Türkiye. Accordingly, geographical location was not used as a stratification criterion, as products sold through these channels originate from centralized distribution centers, rendering city-level supplier information inapplicable. This sampling strategy was deliberately adopted to reflect market-level exposure from widely consumed commercial products, while acknowledging that it may not fully capture variability associated with small-scale local roasters.

These supermarket chains maintain retail networks covering all administrative regions of Türkiye (81 provinces and 923 districts), supported by a nationwide distribution infrastructure exceeding 20,000 physical retail outlets and integrated online grocery platforms. This extensive and centralized distribution structure supports the representativeness of the sampled products in reflecting national consumer exposure rather than region-specific purchasing patterns.

A total of 65 roasted and ground samples were collected between 2021 and 2024 to reflect temporal variability in OTA occurrence, comprising 18 samples in 2021, 18 in 2022, 14 in 2023, and 15 in 2024. All units were sealed packages stored at 4 °C until analysis. The dataset comprised products from 9 nationally distributed brands, with each sample corresponding to a distinct production year and, consequently, to a different production batch. This sampling strategy was deliberately adopted to avoid repeated measurements from the same lot and to ensure that the results represent market-level variability rather than batch-specific contamination events.

All samples were obtained in the most commonly marketed 100 g retail packages, reflecting typical consumer purchasing behaviour. For each sample, brand identity, batch (lot) number, production date, roast degree, and packaging type were recorded to ensure traceability and data integrity. However, brand- and batch-level identifiers were not disclosed to avoid ethical and legal concerns, as the study aimed to assess overall market occurrence rather than brand-specific contamination.

### 5.3. Standard and Sample Preparation

#### 5.3.1. Preparation of Calibration Standards

An aliquot of 0.10 mL of the primary standard solution (10.0 µg/mL in methanol; Trilogy, USA) was transferred into a glass vial and the methanol was evaporated to dryness under a gentle stream of nitrogen at room temperature. The dried OTA residue was then reconstituted in acetonitrile and brought to volume in a 10 mL volumetric flask to obtain a 100 ng/mL intermediate OTA stock/spiking solution. The intermediate solution was stored at −20 °C and remained stable for up to one year.

Working intermediate solutions were prepared by transferring appropriate aliquots of the 100 ng/mL intermediate solution into 10 mL volumetric flasks and diluting to volume with acetonitrile/water/acetic acid (47/51/2, *v*/*v*/*v*). These solutions were subsequently diluted to obtain working calibration standards yielding final concentrations of 0.5, 1, 3, 5, 10, and 20 ng/mL.

#### 5.3.2. Preparation of Samples

For OTA analysis, 5 g of homogenized sample was combined with 100 mL of 1% sodium bicarbonate solution in distilled water. The mixture was stirred for 30 min using a magnetic stirrer (Heidolph, MR Hei-Tec, Schwabach, Germany). Twenty milliliters of the extract, filtered through Whatman No. 4 filter paper, were mixed with 20 mL phosphate-buffered saline (PBS, pH = 7.4) and passed through an immunoaffinity column (OchraStarTM, Romer Labs, Newark, DE, USA), preconditioned with 10 mL PBS, at a flow rate of approximately 3 mL/min. The column was washed with 10 mL distilled water. For elution, 1.5 mL methanol:acetic acid (98:2, *v*/*v*) passed through the column was mixed with 1.5 mL distilled water, yielding a 3 mL final volume collected in 5 mL amber vials. Samples were mixed using a vortex (Heidolph, Reax Control, Germany) and stored at −18 °C prior to HPLC analysis [27].

### 5.4. HPLC Analysis

OTA standards and coffee samples were analyzed using a Shimadzu 10AVP HPLC system (Shimadzu, Milan, Italy) equipped with an RF-10AXL fluorescence detector set at an excitation wavelength (Ex) of 333 nm and an emission wavelength (Em) of 443 nm. NUCLEOSIL Machery Nagel EC C18 (4 × 250 × 5 µm) columns were used at a column oven temperature of 40 °C under isothermal conditions with a mobile phase flow rate of 1 mL/min. Isocratic elution was employed using an acidic (pH 2.8–3.0) mobile phase consisting of water:acetonitrile:acetic acid (47:51:2, *v*/*v*/*v*). An injection volume of 100 µL was applied for all standards and coffee samples. Under these conditions, system pressure was 90–94 bar, analysis time was set at 17.0 min and the OTA peak eluted at a retention time of approximately 14.0 min.

### 5.5. OTA Exposure Assessment

#### 5.5.1. Consumption Data

When the total coffee consumption in Türkiye, reported as 106 million kg, is taken into account, Turkish coffee—assumed to represent 84% of this total—corresponds to approximately 89 million kg per year [33]. Considering that coffee consumption is concentrated within the 15–70 year age group, which comprises 63.6 million individuals in Türkiye [34], the annual per capita consumption of Turkish coffee within this population is estimated to be approximately 1.4 kg per person per year. FAO reports a total per capita consumption of coffee and coffee products in Türkiye of 1.82 kg/capita/year for 2023 [35], which is in good agreement with the Turkish coffee–specific consumption estimates derived in the present study, considering the assumed share of Turkish coffee and population-based calculations.

A portion size of 7 g of ground coffee per cup was assumed, consistent with traditional Turkish coffee preparation practices and the literature [36,37,38]. Based on the estimated annual per capita Turkish coffee consumption of 1.4 kg per person per year within the 15–70 year age group, this corresponds to an average daily intake of approximately 3.8 g per person per day, which is equivalent to about 0.55 cups of Turkish coffee per day.

Accordingly, this estimated average consumption of approximately 0.55 cups per day was used to define the realistic exposure scenario, while 1 cup per day and 3 cups per day were conservatively applied to represent average and high consumption scenarios, respectively, in subsequent exposure and risk assessment calculations. These assumptions are fully consistent with national coffee consumption studies conducted in Türkiye and are assumed to collectively represent approximately 92% of the consumer population [14].

#### 5.5.2. Exposure Assessment

Determining exposure requires detailed food consumption data. In this study, consumption was modelled using three scenarios (0.5, 1 and 3 cups/day corresponding to 3.5, 7 and 21 g/day) to represent realistic, average and high consumers. For exposure calculations, the average body weight of individuals aged ≥15 years in Türkiye (73.5 kg) reported by the Türkiye Health Survey was used. The exposure assessment was conducted using conservative assumptions, assuming complete transfer of OTA from ground coffee to the consumed beverage. Values below the limit of detection (LOD) were treated as the LOD value (0.23 ng/g) for mean calculations and dietary exposure assessment. EDI was calculated using the following formula and expressed in ng/kg body weight/day:

EDI (ng/kg body weight/day) = (Toksin (ng/g)) × (Consumption (g/day))/(Average body weight (kg))

### 5.6. Health Risk Assessment

EFSA has established the provisional tolerable weekly intake (PTWI) for OTA, based on the lowest observed effect level (LOEL) causing kidney damage in pigs, as 120 ng per kg body weight (17 ng OTA/kg b.w./day) [39]. Considering increasing studies on OTA nephrotoxicity, EFSA recommended in 2020 that the MOE approach should replace PTWI for risk assessment [2]. MOE was calculated as follows:

MOE = BMDL10/EDI

Here, MOE was calculated by dividing the BMDL10 (ng/kg b.w./day) by the EDI for probabilistic health risk assessment. The BMDL10 is 4730 ng/kg b.w./day for kidney lesions in pigs (non-neoplastic effects) and 14,500 ng/kg b.w./day for kidney tumor formation in mice (neoplastic effects) [2,40].

### 5.7. Method Validation

The analytical method was validated in terms of linearity, sensitivity, and repeatability. Excellent linearity was achieved for OTA over the tested concentration range, with a coefficient of determination (R^2^) of 0.9998. The limit of detection (LOD) and limit of quantification (LOQ) were determined as 0.23 ng/g and 0.70 ng/g, respectively. The analytical recovery of OTA, determined through spiked sample experiments at concentration levels of 2 ng/g and 10 ng/g, showed a mean recovery of 96%, indicating satisfactory method accuracy. Method precision was confirmed by repeatability experiments, yielding relative standard deviation (RSD) values below 10%, demonstrating the suitability and reliability of the method for the determination of OTA in Turkish coffee samples.

## Figures and Tables

**Table 1:** Amounts of OTA in Turkish coffee samples (ng/g).

2021	2022	2023	2024
OTA (ng/g)	OTA (ng/g)	OTA (ng/g)	OTA (ng/g)
2.25	0.53	2.27	2.1
4.57	<LOD	<LOD	1.84
<LOD	1.02	<LOD	2.05
2.9	<LOD	1.38	<LOD
0.6	0.35	2.22	1.1
1	1.52	0.88	0.92
0.52	0.73	2.51	1.85
0.6	1.24	<LOD	1.55
0.75	1.73	1.12	<LOD
0.93	0.75	1.98	1.2
0.88	19.11	1.45	1.94
<LOD	1.6	1.22	1.28
0.77	1.25	1.1	2.14
<LOD	0.56	2.71	2.4
<LOD	3.91		<LOD
0.89	0.46		
0.58	0.26		
1.03	0.54		
Lowest detected OTA concentration			<LOD
The highest detected OTA concentration			19.11
Average OTA level			1.473

<LOD values were accepted as 0.23 ng/g in mean calculations. Values between the LOD and the LOQ were considered as detected and were used as measured values in the EDI calculations to ensure a conservative exposure assessment. LOD: Limit of Detection; LOQ: Limit of Quantification.

**Table 2:** OTA exposure assessment under three consumption scenarios.

Scenario	C (ng/g)	IR (g/day)	BW (kg)	EDI (ng/kg bw/day)	MOE * (Neoplastic)	MOE ** (Non-Neoplastic)
Realistic (0.5 cup/day)	1.473	3.5	73.5	0.0701	206,847	67,475
Average (1 cup/day)	1.473	7	73.5	0.1403	103,350	33,714
High (3 cups/day)	1.473	21	73.5	0.4209	34,450	11,238

* MOE = BMDL10/EDI; BMDL10 (neoplastic) = 14,500 ng/kg bw/day. ** MOE = BMDL10/EDI; BMDL10 (non-neoplastic) = 4730 ng/kg bw/day. C: Concentration; IR: Intake Rate; bw: body weight; EDI: Estimated Daily Intake.

## Data Availability

The original contributions presented in this study are included in the article/Supplementary Materials. Further inquiries can be directed to the corresponding author.

## Supplementary Materials

The following supporting information can be downloaded at: https://www.mdpi.com/article/10.3390/toxins18020084/s1, Table S1. Summary of market surveys reporting OTA occurrence in coffee products marketed in Türkiye [41,42,43,44,45].

## Abbreviations

The following abbreviations are used in this manuscript:

OTA	Ochratoxin A
EDI	Estimated Daily Intake
MOE	Margin of Exposure
EFSA	European Food Safety Authority
EU	European Union
LOD	Limit of Detection
LOQ	Limit of Quantification
BMDL10	Benchmark Dose Lower Confidence Limit for a 10% response
HPLC	High-Performance Liquid Chromatography
RSD	Relative Standard Deviation
bw	Body Weight
IR	Intake Rate
C	Concentration
