# Supplementary material for: Investigation of Ochratoxin A Levels in Commercially Available Turkish Coffee and Risk Assessment
Source: Toxins (Basel). 2026 Feb 6;18(2):84. doi: 10.3390/toxins18020084 (PMC12944870; doi:10.3390/toxins18020084)
Supplement: Supplementary file 1 [file toxins-18-00084-s001.zip › toxins-4098322-supplementary.pdf]

Table S1. Summary of market surveys reporting ochratoxin A (OTA) occurrence in coffee products marketed in Türkiye.

Coffee type	Sample quantity (pieces)	Positive sample frequency (%)	OTA range (µg/kg)	Method	Year [References]
Turkish coffee	13	100	8.34 - 18.54	ELISA	2015 [41]
Instant coffee	30	36.6	14.01 - 22.54		
Turkish coffee (sold openly)	4	100	9.1-19.2	HPLC	2018 [42]
Turkish coffee (packaged)	6	83.3	1.0-35		
Granulated coffee (sold loose)	4	75	0-0.7		
Granules (packaged)	4	50	0.4-11.0		
Filter coffee (packaged)	12	91.6	0-53.5		
Turkish coffee	90	22.22	0.009-0.053	Immuno-enzymatic	2011 43]
	90	21.11	0.009-0.073	HPLC	
Turkish coffee	44	97.73	0.56-2.6	ELISA	2006 [44]
Instant coffee	25	40	0.135-0.486	HPLC	2020 [45]

References

[41] Yurdakul, O., Sahindokuyucu, F., Yalcin, H., & Keyvan, E. (2019). Survey of Ochratoxin A in coffee, dried grapes and grape pekmez samples in Burdur, Türkiye. *Journal of research in veterinary medicine*, *38*(1), 46-51.

[42] Ergun, B., Soyseven, M., İşcan, G., & Göksel, A. R. L. İ. (2018). Yüksek Performanslı Sıvı Kromatografisi İle Yerel Marketlerde Satılan Kahvelerin Okratoksin A İçeriğine Genel Bir Bakış. *Anadolu University Journal of Science and Technology C-Life Sciences and Biotechnology*, *7*(2), 143-151.

[43] Akçagedik, B. *Kahvede okratoksin a düzeylerinin immuno-enzimatik ve kromatografik yöntemlerle araştırılması* (Master's thesis, Sağlık Bilimleri Enstitüsü).

[44] Metin, R. (2006). *Türk kahvesi örneklerinde okrataksin A varlığı* (Master's thesis, Trakya Üniversitesi Fen Bilimleri Enstitüsü).

[45] Kulahi, A., & Kabak, B. (2020). A preliminary assessment of dietary exposure of ochratoxin A in Central Anatolia Region, Turkey. *Mycotoxin research*, *36*, 327-337.
